# Synthesis of a biomimetic zwitterionic pentapolymer to fabricate high-performance PVDF membranes for efficient separation of oil-in-water nano-emulsions

**DOI:** 10.1038/s41598-022-09046-7

**Published:** 2022-03-23

**Authors:** Nadeem Baig, Zeeshan Arshad, Shaikh A. Ali

**Affiliations:** 1 Interdisciplinary Research Center for Membranes and Water Security, King Fahd University of Petroleum and Minerals, Dhahran, 31261 Saudi Arabia; 2 Chemistry Department, King Fahd University of Petroleum and Minerals, Dhahran, 31261 Saudi Arabia; 3 Interdisciplinary Research Center for Advanced Materials, King Fahd University of Petroleum and Minerals, Dhahran, 31261 Saudi Arabia

**Keywords:** Environmental sciences, Chemistry, Materials science

## Abstract

Oily wastewater from industries has an adverse impact on the environment, human and aquatic life. Poly(vinylidene fluoride) (PVDF) membrane modified with a zwitterionic/hydrophobic pentapolymer (PP) with controlled pore size has been utilized to separate oil from water from their nano-emulsions. The PP has been synthesized in 91% yield via pentapolymerization of four different diallylamine salts [(CH_2_=CHCH_2_)_2_NH^+^(CH_2_)_*x*_ A^−^], bearing CO_2_^−^, PO_3_H^−^, SO_3_^−^, (CH_2_)_12_NH_2_ pendants, and SO_2_ in a respective mol ratio of 25:36:25:14:100. Incorporating PP into PVDF has shown a substantially reduced membrane hydrophobicity; the contact angle decreased from 92.5° to 47.4°. The PP-PVDF membranes have demonstrated an excellent capability to deal with the high concentrations of nano-emulsions with a separation efficiency of greater than 97.5%. The flux recovery ratio (FRR) of PP-**5** incorporated PVDF membrane was about 82%, which was substantially higher than the pristine PVDF.

Oily wastewater is one of the potential contributors to environmental pollution, and it has become a significant concern owing to its adverse impact on the ecosystem. The major contributor to oil pollution is the industries that are not limited to petrochemicals, electroplating, mining, and gas/oil production units^1^. The high demand for oil needs the oil's rapid offshore movement, and nature has witnessed several deadly oil spills. For instance, the Deepwater Horizon oil spill in the Gulf of Mexico has triggered the alarm of the difficulty of the oil–water separation^2^. Owing to its high significance, the development of advanced technologies and methods for the reclamation of the water from the oil-contaminated water has become an area of deep concern. The conventional methods such as adsorption, air floatation, centrifugation, and gravity settling might require high energy and are inefficient in separating oil from water^3^.

To separate oily emulsions, the membranes are considered effective in separating the oil from the water-based on the size-sieving. More control on the pore size of the membranes is required in the separation of the nano-emulsions as they consist of nano-sized oil droplets which easily pass through the rough membranes. Several super-selective materials and surfaces have been reported for the oil/water separation. These materials have been used in the forms of meshes^4^, cotton^5^, foams^6^, sponges, woven/non-woven fabrics^7^. Most of these designed materials effectively separate the floating oils but may face challenges separating the emulsions. Based on the dispersed phase diameter, the different nature of the oil/water mixture can be defined. If the diameter is more than 150 μm, it is termed as free oil and water mixture, while a term of dispersion is used when it is in the range 20–150 μm. The emulsions are generally defined when the diameter is < 20 μm^2^. Due to the complex nature of the emulsions, conventional techniques such as skimming, and gravity separators are ineffective for separating the emulsions. Chemical emulsion breaking may be effective, but high operation costs, significant sludge production, and skilled close control of the system make the overall process disadvantageous^8^. Therefore, more advanced and robust techniques are required for the separations of the emulsions.

Membranes based technologies have emerged as the possible solution to deal with the emulsions owing to their high separation efficiencies and simple operation; as a result, a range of polymeric, glass fiber, and ceramic membranes are designed and analyzed for the oil/water separation^9^. In the modern science of membranes, fouling is one of the key challenges or drawbacks that put serious limitations on using them to separate the emulsions. According to the blocking law, fouling can appear in various forms. For instance, the foulants can sit on the pore openings or penetrate the pores to cause fouling. A cake layer of the foulants can also be formed on the surface of the membrane, which puts serious resistance in the passing of the permeate during separation^10^. In membrane fouling, the membrane chemistry, feed solution characteristics, and operational conditions contribute significantly. Polymeric membranes are famous for separation applications, including polysulfone (PS), polyethersulfone (PES), Polytetrafluoroethylene (PTFE), poly(vinylidene fluoride) (PVDF), polystyrene, and polyacrylonitrile (PAN) membranes. It is interesting to discuss that the polymeric membranes that are relatively hydrophobic or, to a lesser extent hydrophilic have a great tendency to be fouled due to their intrinsic affinity towards the organic foulants and other bio-foulants which adsorb or penetrate the membrane pores^11^. The cake layer formed during operation results in the mass transfer resistance at constant transmembrane pressure, which causes a significant decline in the permeation flux^12^. To overcome the fouling, a range of advanced materials are introduced, including graphene oxide^13^, MOFs^14^, zwitterionic materials^15^, MXenes^16^, and layered double hydroxide^17,18^. Recently, more focus has been observed on the fabrication of selective wettable and self-cleaning antifouling membranes for oil/water separation^19,20^. Similarly, Janus membranes are also introduced with superhydrophobic bottom and a super-hydrophilic top surface for on-demand oil–water emulsion separations^21^.

Among various polymeric membranes, the poly(vinylidene fluoride) (PVDF) membranes are extensively explored and under consideration for separating the oil/water mixtures owing to their excellent mechanical, thermal, and chemical stability^22^. Its processability to fabricate tubular, hollow, and flat sheet membranes have made it advantageous for oil/water separation. Like most conventional polymeric materials, the PVDF membranes are intrinsically hydrophobic, making them susceptible to fouling^23^. Although different hydrophilic nanoparticles are used, aggregation of these hydrophilic particles limits their application in providing the efficient treatment of oily wastewater. It is critical to discuss that a stable hydration layer is required to hinder the oil contact to the surface, which would result in the antifouling behavior of the membranes^24^.

Among various materials, the zwitterionic polymers consisting of motifs of both algebraic signs resulting in an overall neutral charge are considered the advanced materials with immense potential to impart the antifouling properties to the membranes. In several examples, zwitterionic polymers are used to enhance the hydrophilicity of the membranes^25^. Compared to the simple hydrophilic membranes, the zwitterionic membranes have one significant difference of having charges of opposite signs. The simple hydrophilic membranes interact with the water through hydrogen bonding, whereases the zwitterionic polymers interact with the water through the electrostatic interactions. For instance, one of the most famous antifouling materials is poly(ethylene glycol) (PEG) which is usually used in membranes to enhance their hydrophilicity and reduce organic foulant's nonspecific adsorption^25^. The PEG interacts with the water through hydrogen bonding, but the major drawback of the PEG is its vulnerability to oxidation in complex media which results in loosing of the antifouling behavior of the membranes^26^. The change in temperature and the packing density may also cause susceptibility to membrane fouling^27^. Due to this, the zwitterionic materials are considered promising next-generation materials to produce advanced antifouling membranes. In continuation of the efforts, the search for new zwitterionic materials with better compatibility with the PVDF is continued to separate the oil/water emulsions efficiently.

Numerous linear cyclopolymers of industrial importance have been synthesized via free radical polymerization of various diallylammonium monomers [H_2_C=CHCH_2_)_2_N^+^R^1^R^2^]^28–30^. Copolymerization of SO_2_ or maleic acid (MA) with diallylammonium salts having reactivity ratios of almost zero gives alternate copolymers^31^. The polymer backbone, embedded with five-membered rings, is the eighth-most crucial architecture of synthetic polymers^32^. Inspired from the polymer backbone, we synthesized a new zwitterionic pentapolymer (PP) **5** to produce a PVDF membrane with high antifouling performance (Fig. 1). The incorporation of the PP **5** provides an excellent hydration layer that rejects the oil, facilitates the permeation of the water, and significantly reduces the fouling of the PVDF membranes. The incorporation of the PP **5** into the PVDF membrane material has improved the rejection rate substantially and demonstrated the excellent antifouling capability while separating the nano-emulsions.

**Figure 1:**
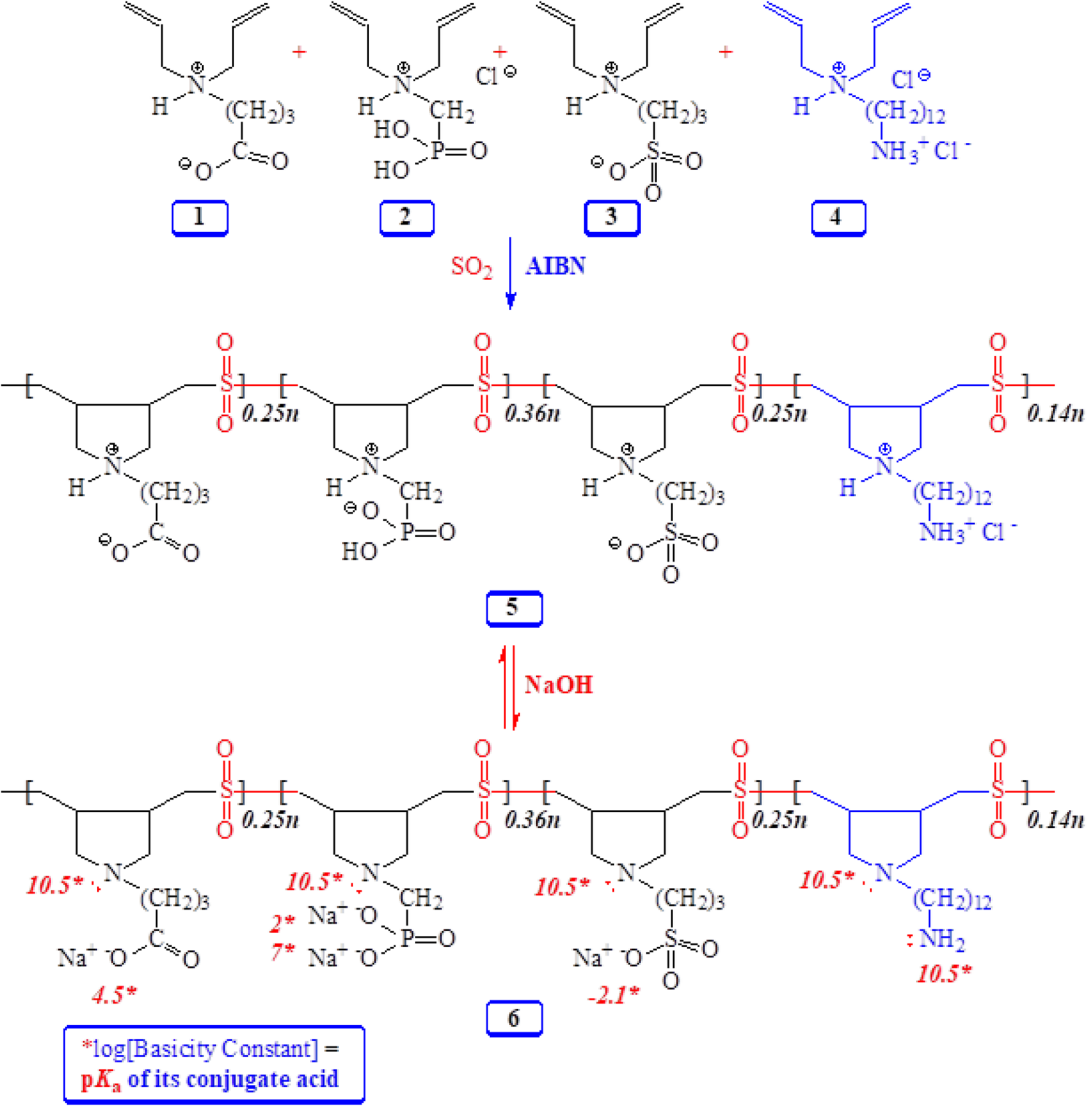
Cycloplymerization of monomers **1**–**4** and SO_2_ leading to PP **5**.

## Materials and methods

### Physical methods

Atomic compositions were determined using a Perkin Elmer instrument (Model 2400). An SDT analyzer (Q600: TA Instruments) was used to perform thermogravimetric analyses (TGA) under a flow of N_2_. The resin's surface morphology was analyzed by Quattro ESEM-FEG-EDX spectrometer (Oxford Instruments) from ThermoFisherTM Scientific. A Thermo Scientific Nicolet iS10 spectrometer recorded the FTIR spectra of the various materials. The lens of the spectrometer was cleaned with isopropanol to avoid any contamination. The PVDF membrane's FTIR spectra were recorded at a spectral resolution of 32 cm^−1^ after 60 scans. Goniometer DSA25 KRÜSS recorded the water contact angle on the surface of the PVDF membranes. Thermo Scientific™ Quattro field-emission scanning electron microscope was used to analyze the surface morphology and the PVDF membrane's cross-sectional analysis. The PVDF membrane's surface and cross-sections were coated with the Au by sputtering to enhance the image quality before FE-SEM images. The liquid nitrogen was applied to cut the membrane to observe the cross-section of the membranes. The Samsung camera was used to record the pictures of the feed and the permeate.

Under N_2_ atmosphere and using CO_2_-free water, the viscosities of solutions of PP **5** were measured by an Ubbelohde viscometer. A Bruker AvanceIII 400 MHz spectrometer was utilized to measure ^1^H, ^13^C, and ^31^P NMR spectra using the residual D_2_O protons (HOD) at δ 4.65 ppm, ^13^C dioxane signal at δ 67.4 ppm as internal standards, and H_3_PO_4_ (85%) in dimethyl sulfoxide (DMSO) as an external standard.

### Materials

2,2′-Azobisisobutyronitrile (AIBN) (≥ 98%) was purchased from Fluka Chemie AG and crystallized from chloroform-ethanol. Dimethylsulfoxide (DMSO) (≥ 99.5%), *N*,*N*-dimethylacetamide (DMA), Bovine Serum Albumin (BSA), and surfactant TWEEN^®^80 were purchased from Sigma-Aldrich. All water used was of Milli-Q quality. A Spectra/Por (Spectrum Lab., Inc.) membrane (MWCO 6000-8000) was used for dialyses. Monomers **1**^33^, **2**^34,35^, **3**^36^, and **4**^37^ were synthesized. The Alfa Aesar-44080 poly(vinylidene fluoride) was purchased with the molecular weight of 350 KDa^38^.

### Synthesis of PP 5

To a solution **1** (1.83 g, 10 mmol), **2** (3.28 g, 14.4 mmol), **3** (2.19 g, 10 mmol), and **4** (1.98 g, 5.6 mmol) in DMSO (11 g) in an RB flask was absorbed SO_2_ (2.56 g, 40 mmol). Initiator AIBN (180 mg) was added to the solution and stirred at 65 °C for 24 h. The thickened mixture (turbid in water) was dialyzed against distilled water for 36 h, during which the polymer solution became turbid and separated as a thick gel at the bottom of the dialysis tube. The whole mixture was freeze-dried to obtain **5** as a white solid (10.3 g, 91%). Note that a HCl unit is depleted from monomer **2** during dialysis to give the zwitterionic motifs in the repeating units of PP **5**. It is calculated that 283.0 mg of PP **5** contains repeating units 0.25 mmol **1**, 0.36 mmol of **2** (-HCl), 0.25 mmol of **3**, 0.14 mmol of **4** and 1 mmol of SO_2_. *v*_max_ (KBr): 3539, 2927, 2858, 1717, 1648, 1461, 1413, 1296, 1205, 1180, 1125, 1033, 906, 747, 668, 603, 536, and 443 cm^−1^. (Found: C, 40.6; H, 6.9; N, 5.3; S 13.6%. PP **5** requires C, 41.55; H, 6.72; N, 5.64; S 14.16%);

### Critical salt concentration (CSC)

The CSC of several salts needed for water-solubility of PP **5** were determined at 23 °C. An aqueous solution (1% wt/wt) of PP **5** in the presence of a salt was titrated with deionized water to turbidity. The concentration of the salt just before the turbidity is the CSC of the salt. The titrations were carried out in triplicate with an accuracy of 1–2%.

### Synthesis of the PP 5 modified PVDF membranes

Before preparing the PVDF membrane solution, the PVDF fine powder was kept overnight under vacuum at 60 °C to remove any adsorbed moisture to avoid its negative impact during the membrane fabrication. The precisely calculated PP **5** zwitterionic polymer was added to DMA solvent to prepare the relevant concentrations for membrane synthesis. The solution was subjected to sonicate for 5 min by probe sonicator to achieve a fine dispersion of PP **5** zwitterionic polymer in DMA. The dried PVDF powder was added into the finely dispersed solution of PP **5** and stirred at 300 rpm at 60 °C for 12 h. The process led to the entire dissolution of the PVDF powder. The PP **5**-PVDF solutions were degassed by the bath sonicator for 30 min and kept for 24 h to make sure that all the trapped bubbles were removed from it. The polymer dope solution was cast on the glass plate with the help of the membrane applicator. The glass plate containing the thin layer of the polymer dope solution was immediately dipped into the coagulation bath. The membrane solidification started immediately as the glass plate dipped into the coagulation bath. The membrane was kept in the coagulation bath for 24 h for complete phase inversion, which helped the maximum leaching of the DMA from the membrane. The membrane's abbreviations, composition, and parameters are tabulated in Table 1.

**Table 1:** Composition of Zwitterionic PP **5** and applied parameters for the synthesis of the various PVDF membranes^a^.

Membrane code	PP 5	PVDF (%)	DMA (%)
M-0	0	20	80.0
M-1	0.1%	20	79.9
M-2	0.25%	20	79.75
M-3	0.5%	20	79.5

^a^Polymer dope solution temperature and coagulation bath temperature were kept at 25 °C.

### Preparation of oil in water emulsion

Oil-in-water emulsions were prepared by adding diesel (1 g) into water (1 L) in the presence of surfactant Tween-80. The mixture was kept under vigorous stirring at 600 rpm at room temperature for 12 h. It was furthermore sonicated for 2 h. The average size of the oil-in-water emulsions was found about 92.71 nm with the help of the Malvern Zetasizer (Fig. S1). The oil/water separation, water flux, and the antifouling performances of the pristine and PP **5**-PVDF membranes were accomplished by fitting the membranes into the dead-end filtration unit. The nitrogen cylinder was used to adjust and supply the appropriate trans-membrane pressure. The membranes were pre-compacted by pure water at a pressure of 8 bar for 1 h. The oil residue in the permeate was determined by the UV–Vis spectrophotometer.

## Results and discussion

### Synthesis and characterization of PP 5

We set out to synthesize a new zwitterionic pentapolymer (PP) **5** (Fig. 1) containing a variety of chelating motifs CO_2_^−^, PO_3_H^−^, SO_3_^−^ and NH_2_ using cyclopolymerization protocol to pursue our planned modification of PVDF membrane for separation of the nano-emulsions. The work is inspired by the zwitterionic phosphatidylcholine headgroups, which contain cationic and anionic groups and are present in the cell membrane phospholipid bilayer. Due to this zwitterionic behavior of the cell membrane, it has fouling-free behavior. Naturally occurring zwitterions, very common in cell membranes, proteins, etc., motivated us to synthesize PP **5** which has pH-responsive zwitterionic motifs conferring charge neutrality, high hydrophilicity, strong dipole pairs, etc. A tightly and stably bounded water layer near zwitterionic polymers via strong electrostatically induced hydration, imparts the antifouling property of zwitterionic polymers by increasing the energy barrier for the adsorption of foulants^39^.

AIBN-initiated cyclopolymerization of monomers **1**, **2**, **3**, **4,** and SO_2_ afforded PP **5** in an excellent yield of 91% (Fig. 1). Since the reactivity ratios of SO_2_ and the diallyl monomers are almost zero, the PP will have the monomers in random distribution alternated by SO_2_. At such a high conversion, the feed ratio is expected to match the monomer incorporation ratio. As such, the ratio of monomers **1**, **2**, **3**, and **4** incorporated in the polymer was taken as 25:36:25:14, respectively. Note that an HCl molecule was depleted from the monomer **2** repeating unit during dialysis to give the zwitterionic phosphonate motifs in the polymer.

Treatment PP **5** with NaOH is expected to generate anionic polyelectrolyte **6**, which has numerous ligands involving N and O, whereby their Log[basicity constants], i.e., the p*K*_a_s of their conjugate bases, range from − 2.1 to 10.5^36,40,41^ (Fig. 1). The amino-carboxylate, -phosphonate, and -sulfonate motifs provide several chelation centers. The pH-responsiveness of the polymer thus provides a greater latitude to trap various metal ions.

**Figure 2:**
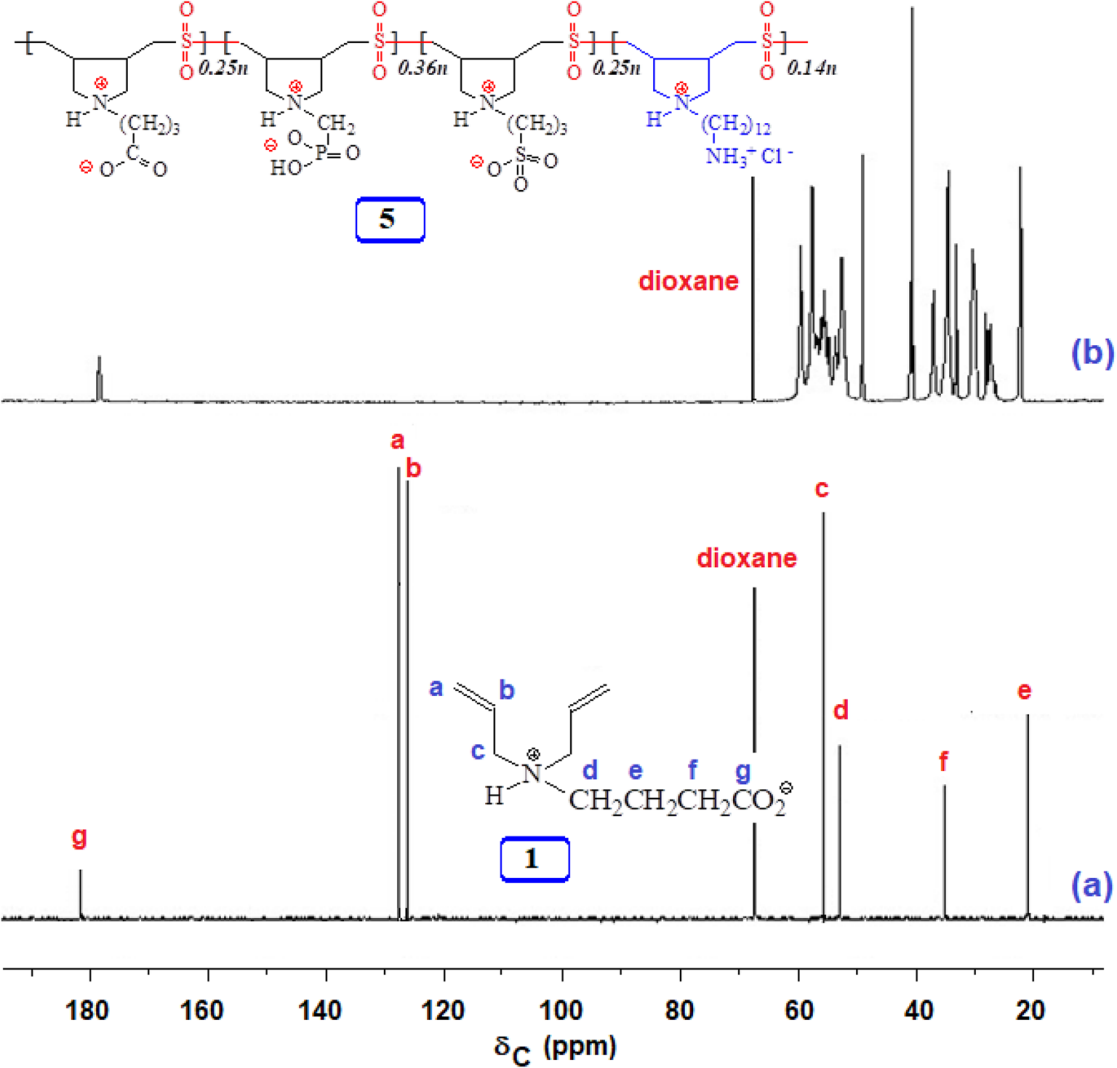
^13^C NMR spectra of **(a**) monomer **1** in D_2_O and (**b**) **PP 5** in 3.5 M KBr in D_2_O.

The ^1^H and ^13^C NMR spectra of PP **5** and monomer **1** is shown in Figs. S2 and 2, respectively. The absence of alkene protons in the range 5–6 ppm and alkene carbons at 127–128 ppm confirms the absence of any residual double bond in the polymer, which suggests degradation chain transfer as the termination process involving allylic hydrogen of the monomers^42^ (Fig. 2). The carboxylate signals at δ182 ppm and ^31^P signals at δ5.72 ppm confirm the incorporation of monomers **1** and **2** in **5**. The elemental analysis supports the incorporation of SO_2_ and SO_3_^-^.

IR bands 1296 and 1125 cm^−1^ were attributed to the SO_2_ groups in PP **5**^43^. The absorptions at 1413 and 1648 cm^−1^ were assigned to the stretching vibrations^44^ of COO^-^. A weak band at 1717 cm^−1^ could be attributed to the CO_2_H owing to the equilibration: –NH^+^(CH_2_)_3_CO_2_^−^ ⇋ –N(CH_2_)_3_CO_2_H. The band at 747 cm^−1^ is assigned to methylene chain (CH_2_)_12_. The IR spectrum indicates the presence of the sulfonate group by its characteristic bands at 1180 and 1033 cm^−1^^43^. The band at 1205 cm^−1^ can be assigned to the stretching frequency of P=O^34^.

It was difficult to obtain molar mass of PP **5** using GPC, since the presence of CO_2_^−^, PO_3_H^−^, SO_3_^−^ and NH^+^ motifs force the polymer to stick to the column materials. A similar observation was found earlier^45^.

### TGA analysis of PP 5

The TGA curve of PP **5** shows a weight loss of 6% up to 200 °C owing to the removal of moisture (Fig. S3). A sharp loss of 23% in the range 220–275 °C is attributed to the decomposition releasing SO_2_; the polymer is calculated to have 22.6 wt% SO_2_. Another major loss of 27% in the range 275–470 °C is accounted for the loss of some of the pendants. The residual mass of 36% remaining at 800 °C could be attributed to some nitrogenous and phosphorous derivatives. The polymer thus remains very stable up to 220 °C.

### Solubility behavior

The water-insolubility of PP **5** is attributed to the effects of interactions of the zwitterionic motifs, which force the polymer backbone to adapt globular conformation^30,46^. The polymer was found to be insoluble in 1 M HCl and 1–5 M NaCl, but soluble in the presence of KBr. CSC value of KBr was determined to be 3.44 M. The stronger binding ability of Br^-^ (as compared to Cl^−^) to the NH^+^ centers can disrupt the zwitterionic interactions and thereby leading to extended conformation and thus promoting water-solubility.

A sample of PP **5** (120 mg) was found to be water-soluble in water (12 mL) in the presence of NaOH (15 mg, 0.375 mmol). The sample (120 mg) is calculated to have repeating units 0.106 mmol **1**, 0.153 mmol of **2** (–HCl), 0.106 mmol of **3**, 0.0594 mmol of **4** (vide supra). Therefore, upon the NaOH treatment, the pH-responsive equilibrium: **5** ⇋ **6** would reside on the right side, whereby the anionic motifs lead to extended conformation and water-solubility.

### Viscosity data

The viscosity plot of PP **5** in the presence of NaOH in 1 M NaCl is shown in Fig. S4. The intrinsic viscosity was found to be 0.0549 g dL^−1^ The low viscosity may be attributed to intramolecular hydrophobic association involving (CH_2_)_12_NH_2_ pendants, which helps to coil up the polymer backbone thereby reducing hydrodynamic volume.

### Characterization of PVDF membranes

#### FTIR and Thermal analysis

**Figure 3:**
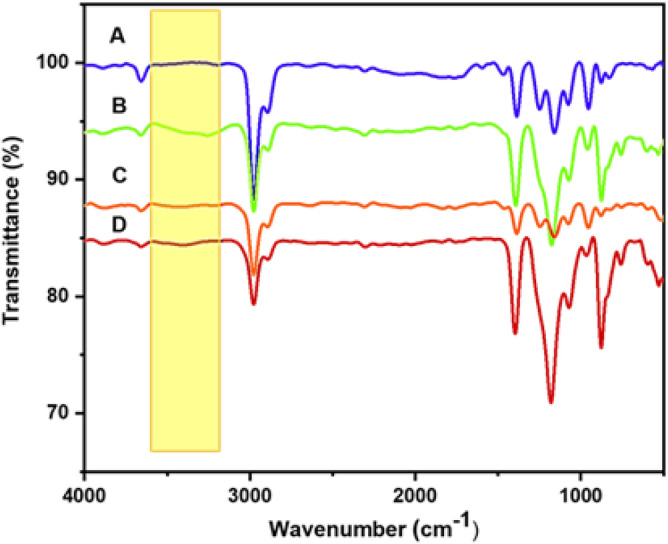
FTIR spectra of (**A**) **M-0**, (**B**) **M-1**, (**C**) **M-2**, and (**D**) **M-3**.

The pristine PVDF and PP **5** zwitterionic polymer doped PVDF were qualitatively investigated with the help of FTIR spectroscopy. The band that appeared at 825 cm^−1^ was assigned to the C–F stretching vibration of PVDF. The PVDF backbone C–C–C asymmetrical stretching vibrations were observed at 877 cm^−1^. A prominent absorption band appeared at 1175 cm^−1^ was assigned to the –CF_2_ symmetrical stretching of PVDF^47^. The fluoro compound generally exhibited strong absorption in the range of 1000 cm^−1^ to 1400 cm^−1^. The characteristics of PVDF β-phase absorption bands observed at 604 cm^−1^ and 1249 cm^−1^ are the fingerprint of the β-phase of the PVDF^48^. The various absorption bands that appeared at 501 cm^−1^, 1077 cm^−1^, 1160 cm^−1^, and 1388 cm^−1^ are the characteristic peaks of the absorption band of the α crystalline phase of PVDF^49^. The PVDF is a fluoro compound, and it has shown strong absorbance in that region, which was evident from the FTIR of the pristine PVDF (Fig. 3A). Due to the –C–H's symmetric stretching, the absorption band was observed at 2887 cm^−1^, whereas the asymmetric stretching was assigned to the absorption band at 2977 cm^−1^^49^. In the case of the PP **5**-containing membranes such as **M-1** to **M-3**, all the characteristic peaks of the PVDF membranes have been observed (Fig. 3). Most of the polymer's absorption bands have appeared very close to the absorption band of the PP **5** and merged with the absorption band of the PVDF. However, the region in which the amino group has been absorbed remained the critical factor in recognizing the successful incorporation of the PP **5** into the PVDF. For instance, in the case of **M-1**, the two partially separated absorption bands appeared at 3246 cm^−1^, and 3340 cm^−1^, and these bands appeared owing to the primary amines present in PP **5**. However, these bands were entirely absent in the pristine PVDF (Fig. 3A).

The thermal stability of the various PVDF membranes was evaluated with the help of thermogravimetric analysis (TGA) in the temperature range 20–800 °C, whereas the temperature was changed 10 °C/min. The pristine or controlled PVDF membranes have shown a sharp loss in weight at 430 °C. The PP **5**-PVDF membranes have shown the decomposition temperature in the range 450–456 °C (Fig. S5). TGA has demonstrated that incorporating PP **5** into the PVDF membranes does not compromise their thermal stability. It is according to the literature that fluoropolymers are more thermally stable compared to polymers consisting of hydrocarbons. The high strength of the PVDF is attributed to the C-F bond's high dissociation energy^50^.

#### Morphological and elemental analysis of PVDF membranes

**Figure 4:**
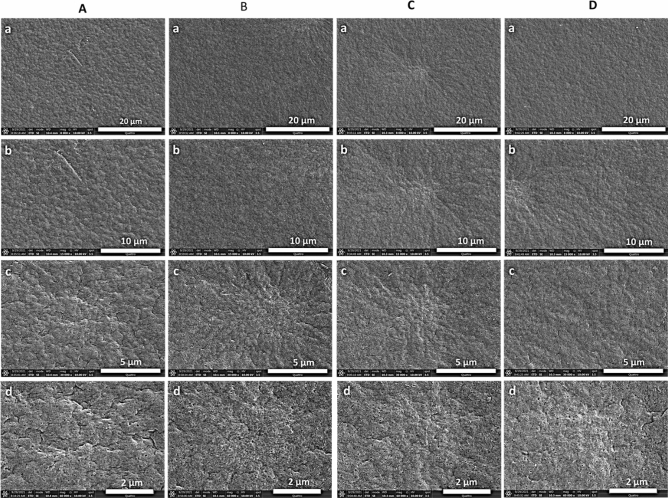
SEM images of the membranes (**A**) M-0, (**B**) M-1, (**C**) M-2 and (**D**) M-3 at various magnifications of (**a**) 8 Kx, (**b**) 15 Kx, (**c**) 30 Kx, and (**d**) 60 Kx.

The surface morphologies of the membranes produced from the 20% PVDF in DMA were scanned with the help of the field emission-scanning electron microscope. Figure 4 presented the SEM images of the PVDF membrane dosed with the different concentrations of the PP **5**. At a lower magnification, the surface of the membranes more a less appeared similar in pristine and PP **5**-PVDF membranes. However, the porosity looks more uniform at higher magnification in **M-1** and **M-2** membranes than in **M-0** and **M-3** membranes. In **M-0** and **M-3**, some pores were observed bigger, which might not be effective in preventing the tiny-sized oil droplets in the nano-emulsions. Later, it was observed that rejection of oil in the permeate of the **M-0** and **M-3** is lower as compared to the **M-1** and **M-2** membranes.

**Figure 5:**
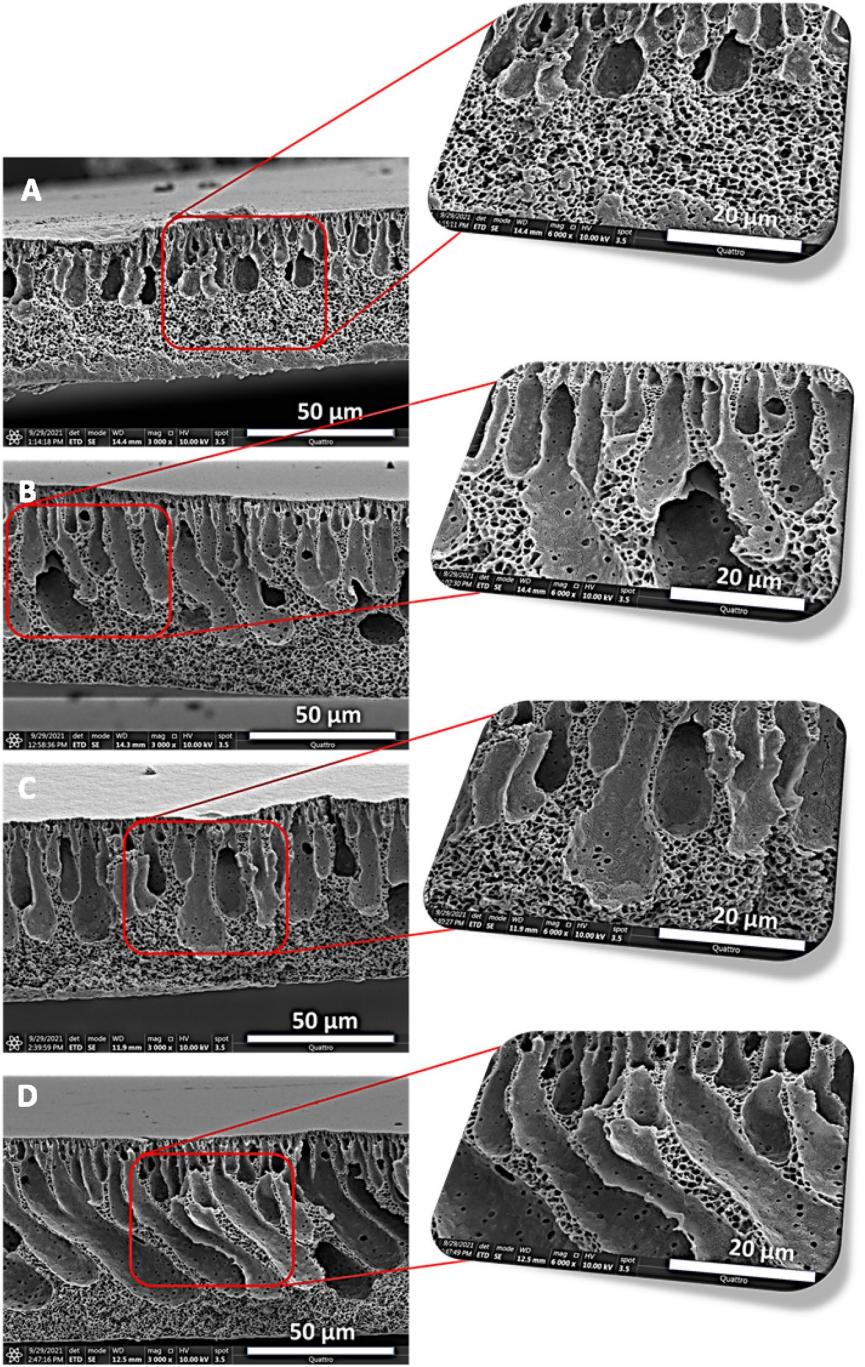
Cross-sectional views of the pristine and mixed matrix membranes (**A**) **M-0**, (**B**) **M-1**, (**C**) **M-2**, and (**D**) **M-3**.

Fabricated pristine and mixed matric PVDF membranes were furthermore evaluated to understand the morphology of the skin layer and the base of the PVDF membranes. The best efforts have been made to preserve the morphology while tearing the membranes for the cross-sectional view. The cross-sectional morphology was preserved by dipping the different membranes into the liquid nitrogen, thereby making the membrane brittle and easily breaking into two pieces by applying a small force. In the cross-sectional view, irrespective of the membranes types, it has been observed that the membranes exhibited an asymmetric structure. All membranes have consisted of the top skin layer, and finger-like projections started from the immediate base of the skin layer (Fig. 5). A typical spongy base was observed in the membranes; however, the length of the dense spongy base varied from membrane to membrane. It is interesting to discuss how the difference in spongy and finger-like structure has been produced in the various membranes after introducing the zwitterionic polymer PP **5**. The high mutual diffusivity of the polymer containing DMA and the water resulted in the formation of the asymmetric structure, and this fact is well known^51^. In the case of the pristine PVDF, the short finger-like projections have been observed, and the rest of the part consisted of the spongy structure. In the case of the mixed matrix membrane, a substantial change in the cross-section of the membranes has been observed. The finger-like projections were grown in breadth and continue in length towards the bottom of the membrane as the amount of the PP **5** was increased in the membranes from **M-0** to **M-3**. The dense skin layer was immediately formed as the polymer dope cast solution immersed into the coagulation bath owing to the rapid out-diffusion of the solvent resulting in the instant solidification of the external membrane surface.

After that, the nonsolvent inward diffusion enhanced, which caused the membrane coagulation and generation of the finger-like projection, which initiated immediately after the skin layer and projected downwards to the base. Interestingly, this effect was more pronounced in the mixed matrix PVDF membranes than the pristine PVDF membrane. This behavior in the mixed matrix PVDF membranes can be explained due to the presence of the hydrophilic groups^52^**.** The water was more attracted inward during the phase inversion process due to the presence of hydrophilic PP **5**, resulting in the enhanced rate of mass transfer between water (nonsolvent) and solvent. The fast mass transfer was due to the presence of sulfonate, phosphonate, carboxylate, and quaternary ammonium motifs, resulting in longer finger-like projections. From the cross-section, it is clear that a spongy structure is present in all of the PVDF membranes. The spongy structure was formed as the polymer solidified, which slowed down the mass transfer between the water and solvent. The spongy structure was more prominent and covered almost more than half of the cross-section in the case of the pristine PVDF membrane. The hydrophobic nature of the PVDF slows down the process, which caused the more dense coverage with the spongy structure. The spongy structure decreased as the hydrophilic PP **5** increased in the PVDF. In the case of **M-2** and **M-3**, the spongy structure substantially decreased, the presence of PP **5** facilitated and kept the sufficient movement of the water and solvent during the solidification of the polymer. This has caused the reduction in the spongy component in the cross-section of the PP **5**-PVDF membranes. It has been shown that incorporating the various concentration of the PP **5** has a significant impact on the subsurface geometry of the membranes.

**Figure 6:**
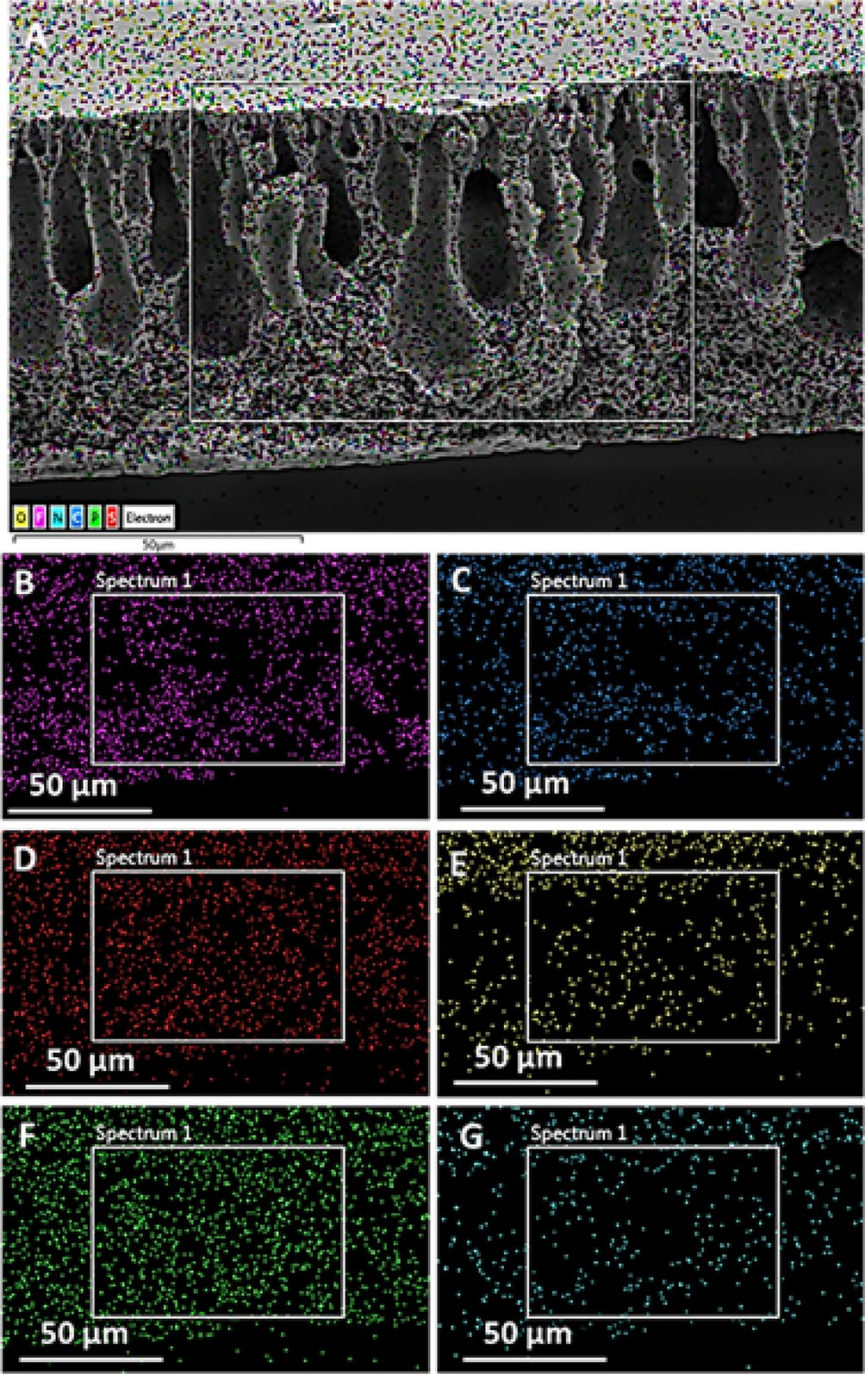
Mapping of PP **5** incorporated PVDF membrane (**A**) complete distribution of elements on the membrane, (**B**) F Kα, (**C**) C Kα, (**D**) S Kα, (**E**) O Kα, (**F**) P Kα, (**G**) N Kα.

As we discussed, PVDF is a well-known hydrophobic material. Sometimes, it is a disadvantage to using it for wastewater treatment as it may show resistance in the permeation of the water. Usually, hydrophilic moieties are used to improve the hydrophilicity of the PVDF membranes^53^. However, the interaction of hydrophilic moieties with the PVDF membranes is poor due to the substantial difference in their surface energy. The PP **5** zwitterionic polymer is more compatible with the PVDF membrane due to the presence of (CH_2_)_12_NH_2_ pendants in PP **5**, which is hydrophobic and provides a better opportunity for its interaction with the PVDF membrane. The elemental mapping has provided critical information about the presence and spread of the PP **5** PVDF membranes. The spread of S, N, O, and P was present throughout the membrane, indicating that the PP **5** spread throughout PVDF (Fig. 6).

### Hydrophilicity of PVDF membranes

**Figure 7:**
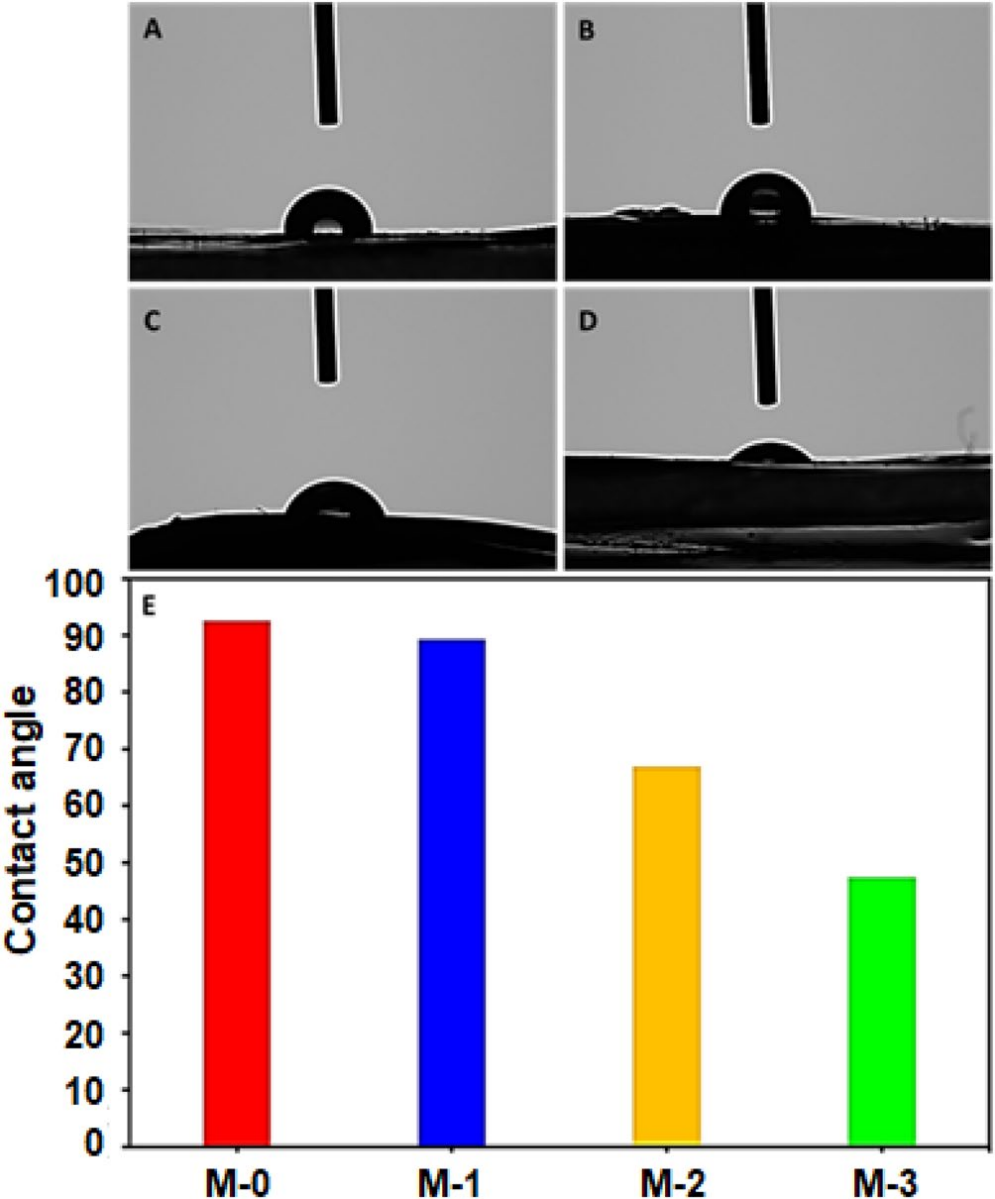
Water contact angle on the surface of the different membranes (**A**) **M-0**, (**B**) **M-1**, (**C**) **M-2** and (**D**) **M-3**.

Pristine PVDF membranes are hydrophobic, which affects their performance during wastewater treatment. Owing to its less attraction towards the water, the optimum performance of the membrane has remained a critical challenge during the separation of the oil emulsified water. The separation performance of the membranes critically depends upon the surface wettability and the appropriate pore size. Surface wettability is one of the significant factors contributing to the efficient separation of the emulsified oil from water. The water contact angle on the surface of the pristine PVDF membrane was 92.5° that has shown that the surface is hydrophobic and not water friendly. This finding is according to the literature^54^. A gradual decrease in contact angle has been observed as the concentration of the PP **5** increased in the PVDF membranes. The contact angle reached 47.4° from 92.5° when the concentration reached up to 0.5% in PVDF (Fig. 7). From the PP **5** structure, it has been clear that it contained a range of anionic and cationic groups such as carboxylate (CO_2_^−^), phosphonate (PO_3_H^−^), sulfonate (SO_3_^−^), and quaternary ammonium motifs. The specifically designed water-loving polymer caused a drastic decrease in water contact angle, and it dropped to 47.4°. Overall, the PP **5** backbone is zwitterionic, and both positively charged and negatively charged groups participated in improving the hydrophilicity of the PVDF membranes, which made them highly effective for the separations of the nano-emulsions (vide infra).

### Separation of oil-in-water nano-emulsions by PP-5-PVDF membranes

Oil and water separation has become critical owing to the rising exploration and industrial applications. Separation of the floating oil is easy, and it can be accomplished by fabricating the super-selective wettable surfaces, which generally have a bigger pore size. The separation phenomena mainly depend upon the selectivity of the surface where surfaces are sharp enough to recognize the non-polar component from water and water from the non-polar oil. However, these sorts of materials sometimes may remain effective for separating the emulsion but, under pressure, might fail owing to the lack of screening potential of the emulsified oil from water. The separation performance has become more complicated when the stabilized oil droplet in emulsions is in the nano range. In the case of nano-emulsion, more control on surface chemistry and pore size is required to separate the nano dispersed emulsified oil drops. The transient fluxes and rejections were recorded at a different transmembrane pressure of 4, 5, and 6 bar. The separation efficiencies of the various PVDF membranes were calculated by Eq. (1)^55^:

1 $$\% {\text{Eff }} = \, \left( {{1} - C_{{\text{p}}} /C_{{\text{o}}} } \right) \, \times {1}00$$

where %Eff is the percentage of separation efficiency, C_o_ is the oil content in the feed, and *C*_p_ is the oil content in the permeate.

**Figure 8:**
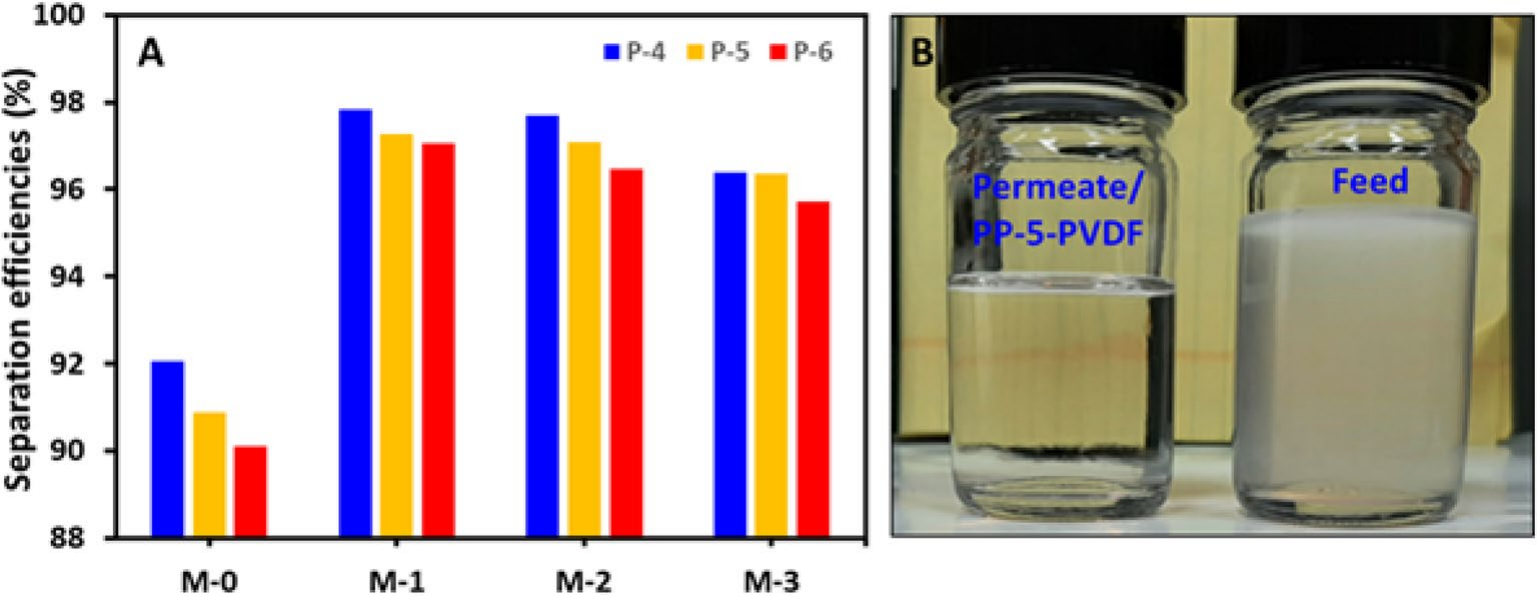
(**A**) Separation efficiencies of the pristine and PP-**5** modified PVDF membranes at different pressure for 1000 ppm oil-in-water emulsion and (**B**) Photographs of the 1000 ppm oil-in-water emulsion (Feed) and collected permeate from the PP-5 modified PVDF membrane.

The pristine PVDF has shown the lowest rejection at all the evaluated pressure of 4–6 bar. The rejection of the pristine PVDF membrane was observed in the range of 90–92%. The introduction of the PP **5** into the PVDF membrane has shown a noticeably positive impact, and the membrane performance was improved substantially. All the PP **5** incorporated PVDF membranes exhibited high performance compared to the pristine PVDF membranes. For instance, the rejection for **M-1**, **M-2**, and **M-3** membranes was observed in the range 97–97.8%, 96.4–97.7%, and 95.7–96.4%, respectively. The highest rejection has been observed with the PP **5**-PVDF named **M-2** and **M-3**, which contained 0.1% and 0.25% PP **5**, respectively (Fig. 8).

A significant impact on the permeation flux of the PVDF membranes has been observed after incorporating the PP **5**. The permeate flux was calculated by Eq. (2)^56^:

2 $$J = \Delta V/A \times \Delta t$$

where *J* is the permeate flux, *V* is the permeate volume in L, *A* is the effective area of the membrane (m^2^) and *t* is the time of the permeation (h).

**Figure 9:**
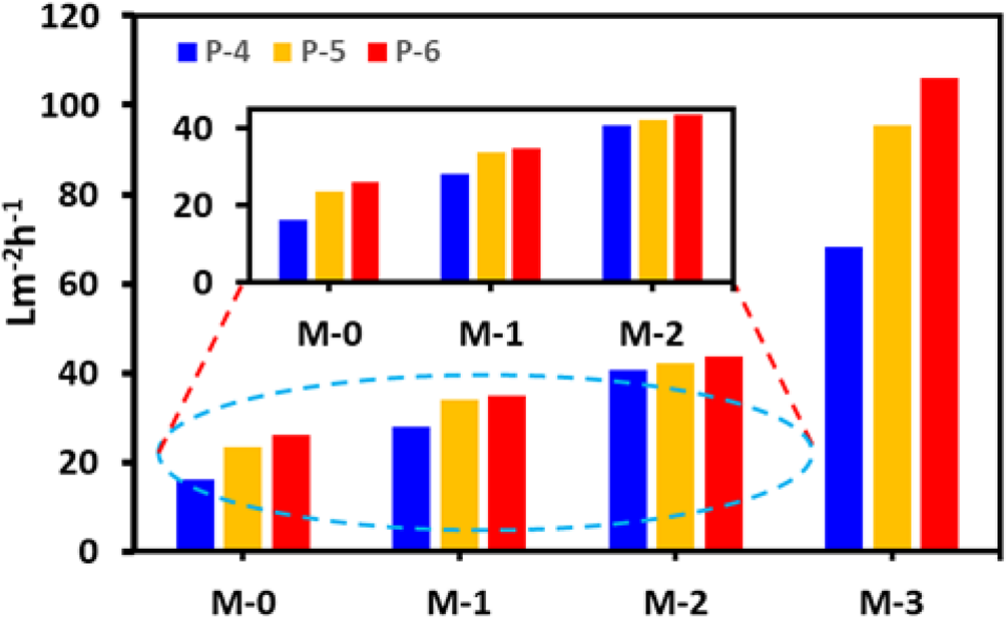
Flux of the membranes (**A**) **M-0**, (**B**) **M-1**, (**C**) **M-2**, (**D**) **M-3**.

At 4 bar pressure, the incorporation of 0.1% PP **5** has shown an increase of 73% of flux compared to the pristine PVDF. The flux increased more than 300% when the doping of PP **5** reached from 0 to 0.5% in PVDF. The permeate flux of the PVDF membrane was in the order of PP_0.5%_-**5**-PVDF › PP_0.25%_-**5**-PVDF › PP-_0.1%_-**5**-PVDF › pristine PVDF (i.e., **M-3** › **M-2** › **M-1** › **M-0**) (Fig. 9). The oil rejection of the membranes was in the order **M-1** › **M-2** › **M**-3 › **M-0**. The permeation flux and rejection are trading off; as the flux increases, the rejection was slightly compromised in the PP **5**-PVDF membranes. As the **M-3**-PVDF membrane (PP_0.5%_-**5**-PVDF) has shown a substantially high flux, its rejection is compromised somewhat compared to **M-1** and **M-2**.

**Figure 10:**
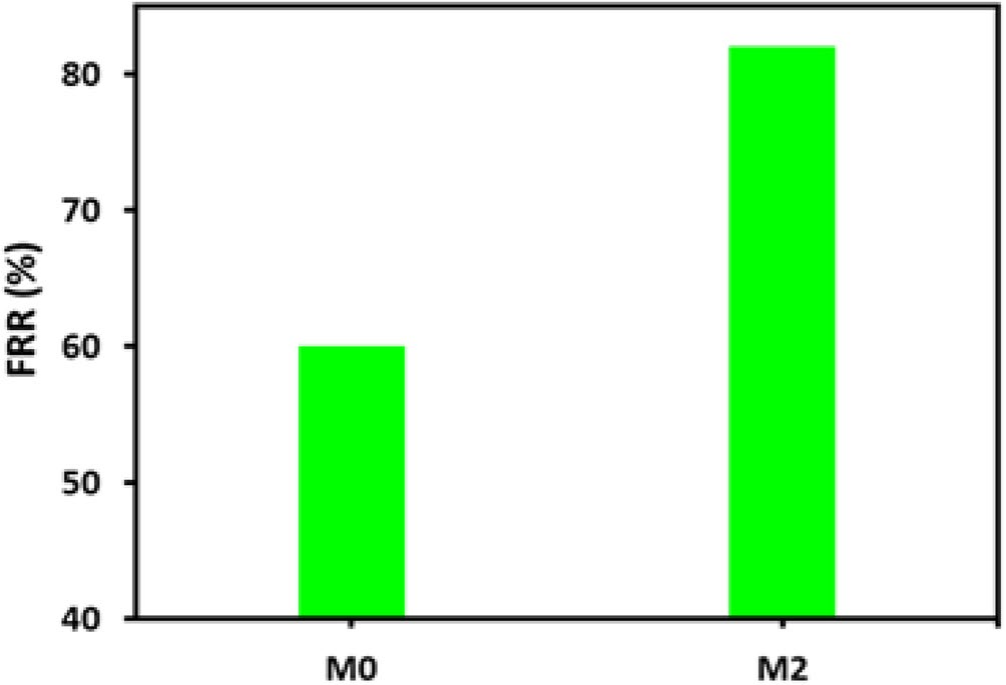
Flux recovery of the pristine PVDF and the PP-**5**-PVDF.

The antifouling behavior of the **M-2** membrane has been further evaluated owing to its appropriate flux and better oil rejection. The membranes were compacted at a pressure of 8 Bar for 1 h, and an antifouling study was performed at a transmembrane pressure of 4 bar. The pristine PVDF (M-0) and PP **5** incorporated PVDF (**M-2**) were exposed to the 1000 ppm oil-in-water emulsion, and after every 15 min, the flux was recorded. After this, the pristine PVDF (**M-0**) and PP **5** incorporated PVDF (**M-2**) were exposed to Bovine Serum Albumin (BSA) solution. For 30 min, it was kept with BSA, and after that, every 15 min, the permeate flux was recorded. The pristine PVDF and PP **5** incorporated PVDF membranes were washed with deionized water to wash out the adsorbed BSA. The flux recovery of the washed membranes was analyzed by exposing them to the emulsions at the same pressure of 4 bar. The Flux Recovery Ratio (FRR) of the pristine PVDF membranes was found to be about 60%, whereas the FRR of the PP **5** incorporated PVDF membrane was about 82% (Fig. 10). The membrane was better equipped to fight against fouling when it was combined with the PP **5**. The results have shown that the pristine PVDF membrane was tough to recover.

### Mechanism of oil-in-water emulsion separation and separation efficiencies

PP **5**-PVDF membranes have shown significant efficiencies for the separation of nano-emulsions. The modified PVDF has demonstrated a considerable capacity to deal with the contaminated water with a high concentration of oil emulsions such as 1000 ppm. The separation of the oil emulsions depends upon several factors, such as membrane pore size, emulsions size, surface chemistry of the membrane. The pore size of the membranes is in the range of nm, so these membranes are quite fit for the separation of the small-sized nano-emulsions. It is evident through their SEM images that the pores sizes are small enough to deal with the emulsions. However, some of the refined oil emulsions passed through the pristine PVDF due to its oil-loving nature, which cannot resist significantly the tiny oil droplets and has shown rejection in the range of the 90–92%. Furthermore, the oil formed a cake layer that resulted in the blockage of the pristine PVDF membrane's pores, resulting in the substantial reduction of the flux during operation. The blockage of the pores or the cake layer was severe in the pristine PVDF membrane that it could not be washed simply by deionized water.

**Figure 11:**
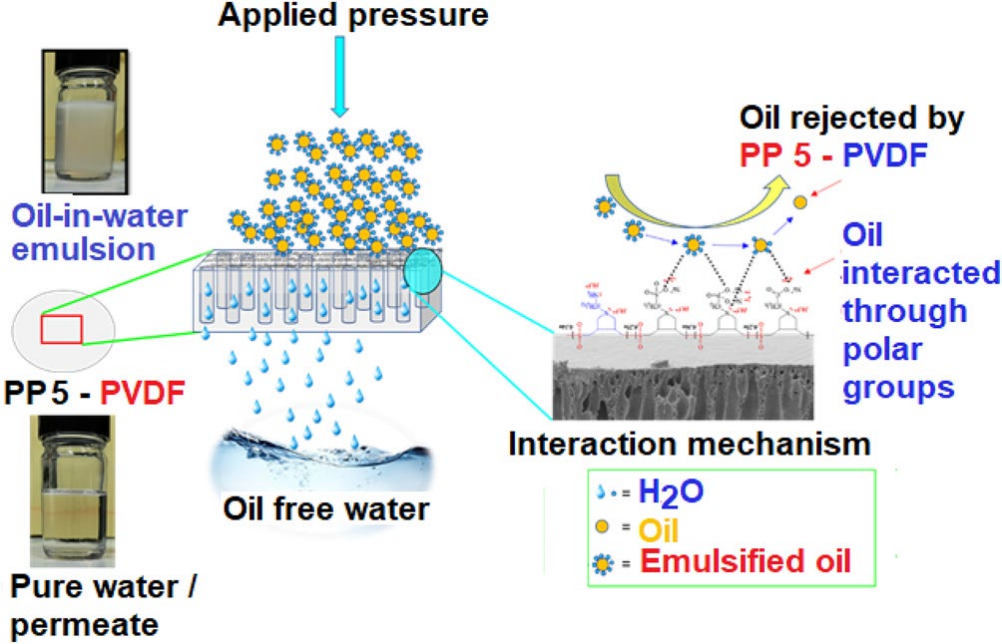
Mechanism of separation of oil-in-water emulsions.

The PP **5**-PVDF membrane was found effective and more efficient for the separation of the oil-in-water emulsions. The pore size of the membrane was refined, which screened the emulsified oil droplet with great efficiency. Furthermore, it has contained a range of hydrophilic functionalities imparted by the doping of the PP **5**. As discussed, the synthesized new PP **5** contained a zwitterionic backbone containing various polar groups such as CO_2_^−^, PO_3_H^−^, SO_3_^−^ and NH_2_. These polar groups imparted the hydrophilicity to the PVDF surface and enhanced the potential to attract the water molecules from the emulsions. The presence of PP **5** assisted in forming the hydration layer, which provided the resistance to the PVDF membrane from fouling from the oil. The zwitterionic polymers are being considered as promising next-generation antifouling materials. The zwitterionic polymers used electrostatic interactions to form the hydration shell. It is a strong interaction between the adsorbed water and zwitterionic polymers, resulting in better antifouling characteristics than the materials where interaction consists of hydrogen bonding^57^. Due to the abovementioned reasons, the water passed easily, oil emulsions broke down, and oil was released and prevented from passing through the PP **5**-PVDF membranes (Fig. 11). The observed permeate appeared as clear water without any emulsion.

The separation efficiencies of some recent membranes used for separation of oil/water emulsions are compared with that of the current membrane in Table 2, which reveals its notable efficacy.

**Table 2:** Comparison of the PP **5**-PVDF separation performance with various membranes.

Membrane	Emulsion concentration (ppm)	Separation efficiencies (%)	Refs
Teflon membrane	5000 to 50,000	86	^58^
TA/DEDAPS-PVDF	1000	>96	^59^
GO/PDA/MCEM	–	96	^60^
Acrylic acid grafted PVDF	–	90	^61^
EGCG-PVDF	1000	94.3	^62^
Ag/EGCG-PVDF	1000	95.4	^62^
PES/PDA@ZnFe2O4 membrane	500	96	^63^
Ceramic (α-Al_2_O_3_) membrane	500	92.4	^64^
Polysulfone membranes	100	> 90	^65^
PVDF membranes	10,000	77	^66^
PP **5**-PVDF	1000	> 97.5	This work

## Conclusions

In conclusion, the de-emulsification of oily wastewater has become critically important to make the water reusable and prevent the collapse of our sustainable ecosystem. Membranes are effective for separating the emulsions, but the separation of the nano-emulsions is a challenging job. In this work, we have synthesized a new zwitterionic polymer PP **5** via cyclopolymerization of diallylammonium salts. The resulting PP **5** contains alternately placed SO_2_ and randomly placed three different zwitterionic motifs of carboxylate, phosphonate, and sulfonate groups. The PP **5** was thoroughly characterized by ^1^H, ^13^C and ^31^P NMR spectra. The PP **5** was incorporated into PVDF membranes to improve their performance for the separations of the nano-emulsions. In PP **5**-PVDF, the finger-like projections have been observed, and the skin layer has shown well-controlled pores in the nanometer range that can efficiently screen the water; and with the help of PP **5**, successfully rejected the nano-sized oil droplets. The TGA analysis has shown that incorporation of the PP **5** did not affect the PVDF membranes' thermal stability, and all the membranes exhibited stability at 400 °C. The presence of polyzwitterionic polymer PP **5** helps in reducing the hydrophobic nature of the membranes and might be responsible for the formation of the hydration layer that assisted in improving the membrane flux and played its role in breaking the oil emulsions. The PP **5**-PVDF membrane was exposed to the feed of 1000 ppm of oil-in-water nano-emulsions and permeate collected that contained just 23 ppm of oil. The PP **5**-PVDF membrane exposed to the BSA and the FRR was found 82%. This study has shown that the zwitterionic polymers have great potential as membrane modifiers. Their presence can substantially improve the performance of the PVDF membranes for the separation of the oil-in-water emulsions.

## Supplementary Information

Supplementary Information.
