# Supplementary material for: Synthesis of a biomimetic zwitterionic pentapolymer to fabricate high-performance PVDF membranes for efficient separation of oil-in-water nano-emulsions
Source: Sci Rep. 2022 Mar 23;12:5028. doi: 10.1038/s41598-022-09046-7 (PMC8943177; doi:10.1038/s41598-022-09046-7)
Supplement: Supplementary file 1 — Supplementary Information. [file 41598_2022_9046_MOESM1_ESM.docx]

**Synthesis of a biomimetic zwitterionic pentapolymer to fabricate high-performance PVDF membranes for efficient separation of oil-in-water nano-emulsions**

**Nadeem Baig^a^, Zeeshan Arshad^b^, Shaikh A. Ali*^b,c^**

^a^Interdisciplinary Research Center for Membranes and Water Security, King Fahd University of Petroleum and Minerals, Dhahran 31261, Saudi Arabia

*^b^Chemistry Department,* *King Fahd University of Petroleum & Minerals, Dhahran 31261, Saudi Arabia.*

*^c^Interdisciplinary Research Center for Advanced Materials, King Fahd University of Petroleum & Minerals, Dhahran 31261, Saudi Arabia*

**Supplementary Figures:**


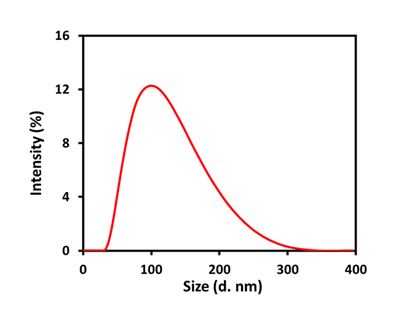


**Figure S1.** Size distribution of the oil droplets in Oil-in-water emulsions


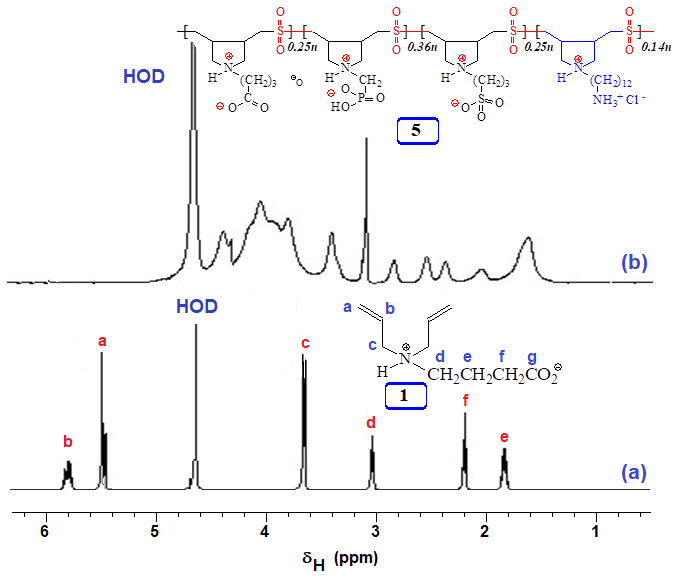


**Figure S2**. ^1^H NMR spectra of **(a**) monomer **1** in D_2_O and (**b**) **PP** **5** in 3.5 M KBr in D_2_O.


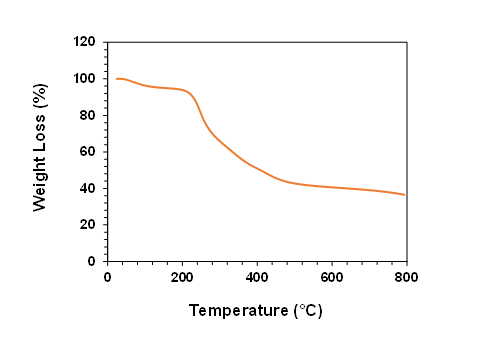


**Figure S3**. TGA curve of PP **5**.


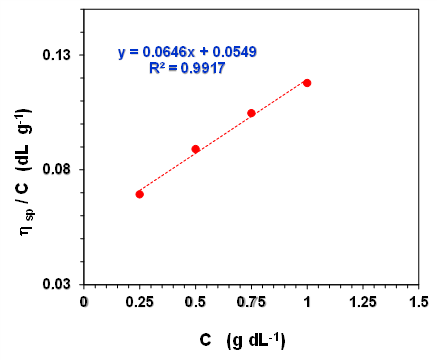


**Figure S4.** Huggin's viscosity plot of PP **5** in 0.1 M NaCl in the presence of 1 equivalent NaOH at 30.0 ± 0.1 °C.


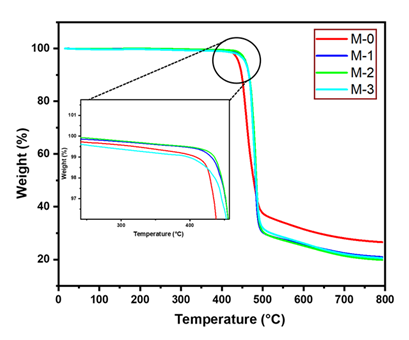


**Figure S5.** TGA curves of pristine and PP-5-PVDF membranes
